# Planning for end of life in the past and present: historical, legal and clinical perspectives on ReSPECT

**DOI:** 10.1177/01410768251317843

**Published:** 2025-02-19

**Authors:** James David van Oppen, Sarah Gunn, Timothy John Coats, Nataly Papadopoulou, Michaela Senkova, Sarah Tarlow, Elizabeth Wicks

**Affiliations:** 1 Centre for Urgent and Emergency Care Research, The University of Sheffield, Sheffield S1 4DA, UK; 2 College of Life Sciences, University of Leicester, Leicester LE1 7HA, UK; 3 School of Psychology and Vision Sciences, University of Leicester, Leicester LE1 7HA, UK; 4 Leicester Law School, University of Leicester, Leicester LE1 7HA, UK; 5 School of Archaeology and Ancient History, University of Leicester, Leicester LE1 7HA, UK

**Keywords:** Emergency medicine, end of life decisions, geriatric medicine, palliative care, ethics, resuscitation

## Abstract

The ReSPECT (‘Recommended Summary Plan for Emergency Care and Treatment’) process was developed in the UK to guide and document conversations and decision-making with patients and their relatives around intervention during critical deterioration. This includes advising whether resuscitation should be attempted when a person dies. Current medical preparation for death is qualitatively different to social behaviours by people in the past and presents some controversies when considering the legal status of death-related decisions. In this article, we discuss our interdisciplinary perspectives as archaeological, historical, legal, medical and clinical psychologist academics following a historico-medico-legal appraisal of the ReSPECT process as situated in the current UK legal and cultural landscape. We review controversies and conundrums, and contextualise and contrast the current position to preparing for death and dying in the past.

## Introduction: the ReSPECT process

Death is universal, but the process of preparing for it is culturally specific. Individuals’ preferences about how and when to die have historically been based on region, religion, class and socially bound tastes, rather than medicine alone. The ReSPECT (‘Recommended Summary Plan for Emergency Care and Treatment’) process indicates an approach to the end of life (EOL), which qualitatively differs from that taken through human history, partly because it addresses choices only possible thanks to modern medical advances.^1^ It frames death as a medical process, aiming for a good death characterised by a balance between the wish to extend the duration of life and our capacity to experience it comfortably.

Before the ReSPECT form was created, there were ‘Do Not Attempt Resuscitation’ (DNAR, DNR or DNACPR) forms, which were locally developed, varied widely and were often not considered valid between healthcare settings. Patients who had a decision not to resuscitate in place were found to be receiving poorer care in settings that were unrelated to resuscitation.^2^ The case for standardised decision recording throughout England was made in a report submitted to the Health Select Committee in 2014.^3^ In response, ReSPECT was developed and first issued by the Resuscitation Council UK in 2016, with the current, third, version of the form introduced in 2020.^4–6^ ReSPECT was designed to support and guide conversations about critical deterioration plans, and to replace DNAR forms with a unified, consistent document to standardise communication about key issues in EOL care.^7^ ReSPECT documents expand on DNAR forms by both presenting additional options of recommendations for normal or modified resuscitation, and by allowing advice regarding treatment escalation thresholds and specific interventions to be recorded. Therefore, ReSPECT was intended to have a broader remit than its forebears, facilitating conversations with all patients and not just those for whom cardiopulmonary resuscitation (CPR) is deemed inappropriate. Both the ReSPECT and DNAR forms differ from Advance Decisions to Refuse Treatment (ADRT), which are legally binding statements by people with decision-making capacity to decline consent to clearly stated treatments in specific situations. These documents and their differences are summarised in Table 1.

**Table 1.** Key differences between ADRT, DNAR and ReSPECT forms.

	ADRT	DNAR	ReSPECT
Primary outcome	Prevent specific procedures or interventions in specific situations	Prevent inappropriate attempts at resuscitation	Provide recommendation for normal, modified or no attempt at resuscitation, and facilitate conversations around other domains related to dying
Validity	Criteria exist for legal validity. Takes precedence over decisions by other people	Typically only valid in the setting in which completed (e.g. community forms were rewritten when people were admitted to hospital)	Ubiquitous and intended to travel with the person between contexts
Rationale for decision	Individual preference to decline consent to clearly stated treatments	Focus on medical justification for anticipated futility of resuscitation	Records the medical diagnoses and prompts exploration of the person’s values and preferences
Personal capacity	The person must have decision-making capacity	Typically documented but not necessary for decision	Intended for completion with active involvement, but the decision can still be made where the individual does not have the relevant decision-making capacity
Involvement of relatives	The person is advised to inform next of kin	Relatives required to be notified. Recording on forms varies from checkbox to narrative entry	Document prompts discussion of form contents
Decision maker	The person. For refusal of life-sustaining treatment, a witness signature is required	Clinician	Clinician, although the form prompts involvement of the person
Contact information	Typically not included	Typically not included	Records emergency contact details

ADRT: advance decision to refuse treatment; DNAR: do not attempt resuscitation; ReSPECT: recommended summary plan for emergency care and treatment.

The ReSPECT form is the written product of an intended broader ‘ReSPECT process’, aimed at supporting creation of personalised recommendations for clinical care and treatment in a future emergency when an individual cannot make or express choices. The completed form is intended to travel with the patient, and completion and documentation of the decision is clearly badged as one stage in a larger conversation.^8^ Guidance for healthcare professionals (HCPs) from the Resuscitation Council UK’s website around ReSPECT conversations recommends reaching a shared understanding of the person’s current health and how it may foreseeably change, identifying what matters to the person regarding care goals in any future emergency, and using that to record an agreed focus of care, including shared recommendations about specific treatments.^9^ This goal is demanding and these conversations can be uncomfortable, being the only time in medicine where a futile option must be discussed with, or even offered to, a patient. How recommendations are ‘shared’ with patients is ambiguous.

Gaps exist between the intended use of ReSPECT and its real-world application. Specifically, ReSPECT appears to be considered more a clinical process around EOL care than a person-based unified plan for future deterioration.^10–12^ ReSPECT has been implemented in around two-thirds of UK hospitals and around half of general practice settings. It is predominantly completed with older people, and recommendations are typically not to attempt resuscitation.^13–15^ Experts have also highlighted shortcomings in clinical consultation skills, written communication and interpretation of these documents.^16,17^ Since the ReSPECT form prompts clinicians to discuss and record patients’ priorities concerning EOL care, questions are raised about its legal status and its relationship with other records such as advance decisions and ‘end of life plans’.

In this article, we evaluate ReSPECT clinically, legally and historically. We begin by considering current controversies regarding the position of ReSPECT in UK legal and clinical perspectives. We then contextualise concerns and conundrums in current UK attitudes to preparing for death with historical Western European social traditions and norms. Our ethical perspective prioritises respect for patient autonomy at EOL, while recognising practical challenges faced by clinicians within emergency and other critical settings. We invite readers to consider historical and social traditions and norms, while reflecting on modern medical advances, medicalisation and secularisation of death, expectations of clinicians versus realities of healthcare practice and the role of the law in the context of preparing for death. We note that there is not just one way of approaching or treating death, and more awareness is needed of past practices, legal options and clinical realities.

## Practical issues in clinical practice: recording ‘shared recommendations’

There are undoubtedly both practical and legal difficulties around achieving the admirable goal of recording shared recommendations. One overarching concern with the ReSPECT form is a lack of clarity around its intended audience. In places, it uses the words ‘my’ and ‘me’, suggesting that the patient (or their representative) should read and complete the form. For example, section 2 is labelled ‘shared understanding of my health and current condition’ and section 3 focuses on ‘what matters to me in decisions about my treatment and care in an emergency’. However, other sections are clearly for clinicians’ completion, and the form only requires a clinician’s signature. This seems a significant missed opportunity to ensure, and record, the genuine involvement of the individual, which is vital for demonstrating compliance with the Mental Capacity Act 2005 (MCA). Should legal issues or disagreements arise in future, the absence of proof that anyone other than a clinician had been involved in completing this form could be problematic. Accordingly, from a pragmatic perspective, inclusion of the individual in a formalised sense could be mutually beneficial.

In practice, there are further issues around meaningful patient involvement. While many forms are completed with careful shared reflection and planning as intended, it is also commonplace for ReSPECT completion to be undertaken in an emergency setting with a patient lacking capacity, when a treatment decision must be made urgently. For example, EOL care forms such as ReSPECT might be hurriedly completed in the emergency department when a person appears to be imminently dying, or completed just prior to emergency surgery when the person is distressed and in pain. In these contexts, documents may be misperceived as being part of a process or routine that clinicians are, or feel, obliged to follow. While such hurriedly-completed forms provide a record of unnecessary interventions being prevented, rushed documentation may not articulate the rationale for recognition of futility or decisions, and are not necessarily a record of shared decision-making in the way intended. It is, however, crucial to acknowledge the wider medical, legal and systemic issues that contribute to these rushed completions at times of necessity, rather than preparation of these key documents being undertaken well before they are needed.

Many ReSPECT forms are also completed in the community with people lacking decision-making capacity, especially those living with frailty. In this context, a 2021 focus group study of GPs concluded that ‘*Conceptualising ReSPECT as an end-of-life care document suggests a difference in how general practitioners understand ReSPECT from its designers*’.^11^ The form requires that if an individual has no capacity to participate in making recommendations, then a ‘ReSPECT conversation’ must take place with ‘the family and/or legal welfare proxy’. In this scenario, the concept of ‘shared’ understandings and recommendations takes on a subtly different form. From the perspective of the MCA and the need to consider, and indeed prioritise, the wishes and feelings of the person lacking capacity, it is important for the ReSPECT conversation to also include that person if possible.^18^

The aims and objectives of the ReSPECT process are well-known among HCPs. However, these appear difficult to achieve when the ReSPECT is used in emergencies or where people may lack capacity. When the process that the form intended to guide and document has not been fulfilled, we are led to consider possible limitations of compatible applications in busy or complex situations. Overall, these limitations point to the need for a wider societal, legal and clinical shift towards the routine consideration of EOL decision-making earlier in life, with regular review of wishes as the individual moves through their life.

## Legal context of the ReSPECT form

ReSPECT clearly states that the form itself is ‘not a legally binding document’. However, this is over-simplistic from a legal perspective. It raises questions of why the recorded ‘shared recommendations’ are not regarded as legally binding, and what the purpose of the ReSPECT form is intended to be, if not to provide legal protection and justification for future care and treatment decisions.

There are already ‘legally binding’ documents in the UK for people to use to plan for critical deterioration. An ADRT is, under Section 26 MCA 2005, explicitly binding just as a contemporaneous refusal.^19^ ADRT allows people to state their refusal of consent to specific interventions in defined situations, before a deterioration in health that renders them unable to articulate that preference. There is a prompt within the ReSPECT form to include details of ‘other relevant care planning documents and where to find them’. This provides a valuable opportunity to highlight an existing ADRT, and could prompt initiation of a new ADRT if the person involved retains capacity. Within the ReSPECT conversation, it would be ethical and judicious for HCPs to direct eligible people towards drafting an ADRT if that accords closely with their future wishes. Provided it is valid and applicable, ADRT should take priority over any other relevant documentation.^20^ Its legal status therefore offers security and certainty for individuals, who are protected from procedures that they do not wish to receive.

However, ADRT is only available to a person who retains decision-making capacity under Sections 2 and 3 of the MCA.^19^ It is also unlikely to be drafted in practice in emergency situations. If a person already lacks decision-making capacity, then they cannot state an ADRT and the ReSPECT form is the only way to register their views and priorities. This is significant, given that in the UK people might discuss but not truly deliberate on dying until its process has begun, and that for a proportion of people at this stage of life it is too late to document their healthcare goals in a legal ADRT. The current legal structure, accordingly, makes certain people who are dying ineligible for the protections of ADRT despite having perhaps discussed their preferences with friends and loved ones.

MCA Section 1(5) makes clear that all decisions made in respect of a person who lacks capacity must be made in their best interests.^19^ The courts have been increasingly clear about the importance of determining a person’s best interests on a subjective basis, as opposed to deciding what appears objectively reasonable. There is a requirement for HCPs to consider not just the treatment and likely outcome, but also carefully deliberate the individual’s likely attitude to these, given their social and psychological situation.^18^ Given that MCA Section 4(6) explicitly requires consideration of the person’s ‘past and present wishes and feelings’ as well as ‘the beliefs and values’ and other factors that would be likely to influence that person’s decision-making, the ReSPECT form should surely be considered an eligible legally binding record of these for future best interests determinations.

Where a ReSPECT process results in a decision not to recommend attempting resuscitation, then a legal duty would exist to respect the right of consultation for the patient and/or their family. The courts have been clear that there is a presumption under Article 8 European Convention on Human Rights (ECHR) in favour of involving a patient and/or their family in a decision whether to place a DNAR notice on their hospital notes.^21^ In *Tracey*, there was held to be a procedural violation of Article 8 ECHR when a DNAR notice was placed on a patient’s hospital notes without consulting or notifying the patient or her family about the decision that CPR was not in her best interests. In the later Winspear case, this principle of consultation was extended to the family of an adult who lacked capacity.^22^ This requirement for patient/family involvement in treatment decisions is clearly denoted within MCA Section 4(7), which states the need to consider the wishes and feelings of the adult lacking capacity, and to consult ‘if it is practicable and appropriate’ with ‘anyone engaged in caring for the person or interested in his welfare’.^19^ Failure to do so means that a ReSPECT form recommending DNAR will be procedurally flawed under the MCA, which may amount to a procedural breach of Article 8 ECHR.

Beyond ADRT and DNAR notices, other decision records might also exist alongside a ReSPECT form such as lasting powers of attorney, mental capacity assessments and best interests determinations. It is presently unclear how the ReSPECT process relates to these existing legal and ethical processes. The stated aim of reaching ‘shared recommendations’ seeks to capture both the patient’s values and the clinician’s judgement of prognosis, but whether that can be comprehensively achieved and these synthesised into concise and robust recommendations for future care remain open to question. From a legal perspective, there are (at least) two tasks that the ReSPECT form seeks to accomplish: serving as a checklist to ensure doctors consider individual circumstances before making clinical recommendations, and providing a means by which a patient and/or their family can express their wishes regarding future care. Both tasks have meaning in law. At the time of critical deterioration, it is clearly desirable to have a summary of situation and plan readily available in a short and concise document. In life-threatening situation decision-making, however, a clinician may need to consider one or more of the following considerations: (1) a clinical assessment that specifies that future treatment will not be in the patient’s best interests; (2) expressed capacitous wishes about future treatment, which may be binding if amounting to a refusal of treatment; (3) expressed non-capacitous wishes and feelings of relevance to a best interests determination; and (4) the views of the family, which are also a relevant consideration in establishing best interests under the MCA. Using the ReSPECT form to fulfil these four considerations renders it a highly complex document. The deliberation, discussion, rationale and eventual recommendation in following the ReSPECT process will likely have been recorded in longer narrative form in medical records, and these will inevitably be unavailable at the time the form is needed, when typically there is very limited information to hand. The protection afforded to those following summary recommendations has not yet been tested in case law.

To summarise our legal appraisal, the ReSPECT form provides an important additional means of recording and ensuring respect for a patient’s choices, values or priorities, and from a legal perspective is a relevant document when making treatment decisions. While it is technically correct that the form is not ‘legally binding’, it is nonetheless a highly relevant piece of documentation in the determination of lawful treatment decisions. Indeed, it is hard to see how a treatment decision about a person who has a ReSPECT form could comply with the MCA unless the form is considered. Due to their desirable brevity and portability, there is a limit on the detail and specificity of ReSPECT form recommendations, and decision-making based on these has not yet been challenged or tested in law.

## Contextualising the current situation: historical viewpoints on planning for death

The recommendation options within ReSPECT in part respond to a default assumption in modern Global North societies that, in the absence of a compelling case to the contrary, extension of life is good. This idea is found in law in the right to life and similar provisions, and in the default stance to resuscitate within clinical guidance.^23,24^ Artificial extension of biological life was typically impossible before the mid-twentieth century.^25^ Before that period’s pioneering emergence of chest compression and mechanical ventilation, people did not necessarily view early death as negative or undesirable.

Beliefs about what constitutes a ‘good death’ are historically variable and also understood differently by major world religions. In recent debates about assisted dying in the UK, Christian and Muslim groups have mostly opposed human intervention to end life, which is often considered sacred. However, human intervention in favour of life’s artificial prolongation has also been seen traditionally as an arrogant appropriation of divine power. Think, for example, of the way that Mary Shelley presented Frankenstein’s attempts to bring a man back from death as fundamentally opposed to the will of God.

A full survey of religious attitudes to EOL is beyond the scope of this article (however, see literature^26,27^). It is worth noting here that beliefs about death are not restricted to adhesion to a religious orthodoxy. Complex, secular beliefs are often held in parallel with both scientific and religious ones. In modern, Western society, such beliefs might include ghosts, angels, reunion with loved ones and potent metaphorical understandings such as going to sleep, held even by people who do not consider themselves to follow a particular faith.

Taking into account not only medical and legal perspectives, but also religious and cultural ones is a powerful corrective to unquestioned assumptions about what a good death looks like. In this respect also, a review of practices and attitudes from the classical and historical past can be instructive.

Numerous historical examples show that abbreviating life was considered not only acceptable, but honourable or even expected, contrasting with many cultures today where enabling suicide under any circumstances remains controversial.^28^

Literary sources from classical antiquity are replete with stories about individuals and groups who actively pursued death, or accepted premature dying because the sociocultural situation demanded it. The alleged Spartan tradition of not surrendering in battle is a well-known example. Such practice was encouraged by its idealisation in Tyrtaios’ poetry in the seventh century BC and inspired one of the most famous examples of Spartan self-sacrificial military behaviour at the battle of Thermopylae in 480 BC. The Greek historian Herodotus describes the fates of two Spartan survivors. The first chose to die by suicide after returning to Sparta because of unbearable social excommunication (Herodotus 7.232). The other redeemed himself by actively pursuing death at the hands of enemies in the battle of Plataea the following year (Herodotus 7.229–31). Both men surrendered their lives prematurely because of sociocultural pressures, hoping to retrieve their honour. Their deaths were seen as appropriate in timing and execution according to Spartan values.

Premature death might have been seen as something to embrace, enjoy and celebrate even in the civilian setting. The modern term euthanasia, which literally translates to ‘good death’, originally described just that – a joyful dying process regardless of life stage or medical condition of the person experiencing it. When first used, the term was linked with scenes of personal enjoyment and expressed a poetic wish to die on the spot when the person was most happy. The Greek comedy writer Menander (fourth century BC) vividly declared his wish for a ‘good death’ (euthanasia) after a lavish meal, the point when he felt most content (Fragment 23).

Another classical author reports a law allegedly followed by communities on the Greek island of Ceos, according to which anyone reaching age 60 was compelled to drink hemlock so that resources may be ‘sufficient for the rest’ (Strabo 10.5.6). The validity of this anecdote and its specific historical period are unknown. There are stark differences between such involuntary euthanasia and neoliberal constructs of individual autonomy. The tale was, however, complemented by attribution of an improved recipe for preparing hemlock for this purpose, which apparently aimed to make death ‘swift and easy’ (Theopharstus, *Enquiry into Plants* 9.16.9). This emphasises the determination of the Ceos people to tame, organise and manage the dying process by making it as comfortable as possible, given the means available.

**Figure 1.**
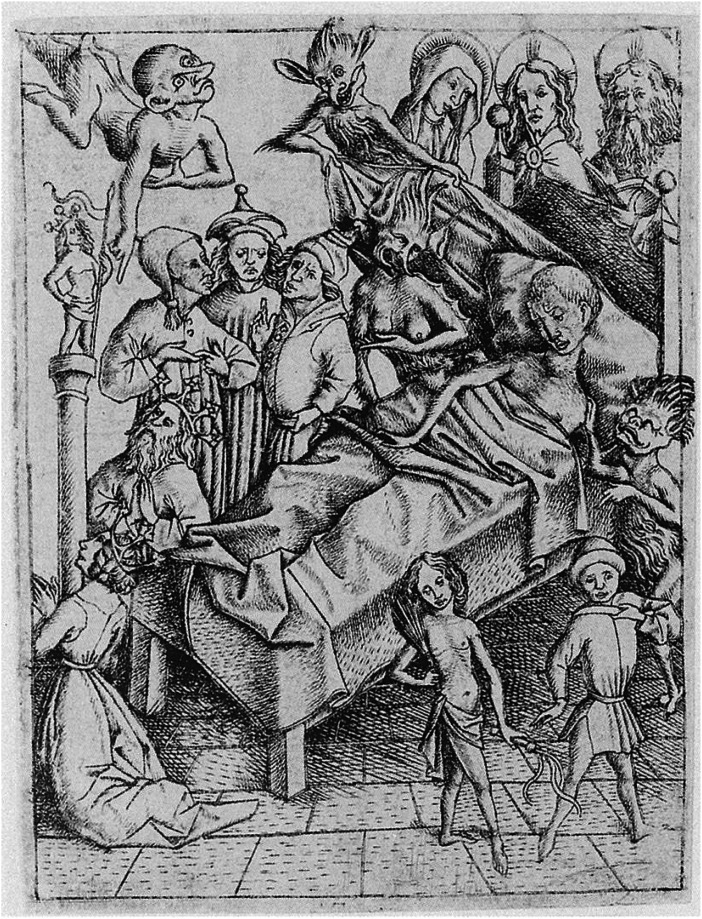
Image taken from the 1450 edition of the Latin *ars moriendi*, which was widely translated and distributed around Europe. ‘Lack of faith’, part of a series depicting deathbed temptations. Note the large number of human and supernatural beings present at death., Wikipedia en. By Master E. S. - Janez Höfler: Der Meister E.S: Ein Kapitel europäischer Kunst des 15. Jahrhunderts., Tafelband, Schnell & Steiner, 2007, ISBN 978-3795420277, L.175, Public Domain, https://commons.wikimedia.org/w/index.php?curid=4518382

Comparing contemporary texts of dying (such as the ReSPECT form) and late medieval/early modern *ars moriendi* (art of dying) literature is also illuminating. This is first because death now involves making choices and decisions. Former medical technology offered fewer solutions, and therefore it was rarely necessary to decide whether interventions were appropriate. Similarly, there were few available options requiring decisions around balancing the duration of life and optimising its quality. Nor, until recently, would medical practitioners necessarily be involved in the process of dying. Dying was not a failure requiring investigation and explanation of what went wrong; it was an inevitable part of every life. The woodcut illustrations of crowded deathbed scenes printed in medieval *ars moriendi* texts included relatives, neighbours, clerics, angels, sometimes even a lawyer to take care of material matters, but not a doctor (Figure 1). The fact of death was omnipresent, and for most people, preparing for death was a lifelong project shared with their community.

The ReSPECT form invites modern people to do something similar – to reflect on the dying process and discuss what matters to them, perhaps thus reclaiming dying from medicalisation as a normal part of life.^29^ When conducted as intended, ReSPECT discussions consider the person’s values and perspectives, and a clinical recommendation for care. However, whereas the *ars moriendi* literature was vividly present throughout a medieval person’s life, and discussion about how best to end one’s life was a normal part of the discourse of young and healthy people, the ReSPECT form is often introduced at a moment of crisis, as a way of discussing an otherwise distasteful and often taboo subject. The authors of *ars moriendi* texts would have agreed with the Lancet Commission on the Value of Death that discussions of how to make a good end should be normalised among the young and healthy.

## Summary

Although the medical organisation of death is mostly specific to the modern age, attempting to plan and control one’s death was widely practised in the past. The ReSPECT process addresses two aspects of dying: comfort (especially avoidance of pain) and duration of life. Of these, only the avoidance of pain was potentially achievable through most of human history, and even that was not always considered an important part of dying. Most of our ancestors were more willing than we are to recognise death as inevitable, though they did approach it with other values that were culturally specific. Meanwhile, modern approaches to death and dying reflect a society that values individual difference, unlike many past societies in which culturally normative practices downplayed differences in personality, philosophy or values. The ReSPECT form is part of a late modern project to tame, organise and manage death, perhaps imperfectly executed in a complex legal and clinical landscape, but driving towards inclusion and shared planning between the patient and the clinician. The need to integrate personal values with clinical recommendations prompts reflection on the best way to undertake and record shared decision-making about the EOL. A deep-time perspective makes it clear that death is not inevitably the proper concern only of HCPs. The ReSPECT process, thoughtfully instituted, is an opportunity for medicine to operate in the service of society, culture and individual people, in making genuinely consultative decisions.

## Declarations

### Guarantor

JvO.

### Provenance

Not commissioned; peer-reviewed by Sarah A Hopkins and Bruno de la Villéon.
